# Urbanism and the division of labour in the Roman Empire

**DOI:** 10.1098/rsif.2017.0367

**Published:** 2017-11-15

**Authors:** J. W. Hanson, S. G. Ortman, J. Lobo

**Affiliations:** 1 Department of Anthropology, University of Colorado, Boulder, CO, USA; 2 School of Sustainability, Arizona State University, Tempe, AZ, USA; 3 Santa Fe Institute, Santa Fe, NM, USA

**Keywords:** complex systems, settlement scaling theory, urbanism, division of labour, Roman Empire, economic development

## Abstract

One of the hallmarks of human agglomeration is an increase in the division of labour, but the exact nature of this relationship has been debated among anthropologists, sociologists, economists, and historians and archaeologists. Over the last decade, researchers investigating contemporary urban systems have suggested a novel explanation for the links between the numbers of inhabitants in settlements and many of their most important characteristics, which is grounded in a view of settlements as social networks embedded in built environments. One of the remarkable aspects of this approach is that it is not based on the specific conditions of the modern world (such as capitalism or industrialization), which raises the issue of whether the relationships observed in contemporary urban systems can also be detected in pre-modern urban or even non-urban systems. Here, we present a general model for the relationship between the population and functional diversity of settlements, where the latter is viewed as an indicator of the division of labour. We then explore the applicability of this model to pre-modern contexts, focusing on cities in the Roman Empire, using estimates of their numbers of inhabitants, numbers of documented professional associations, and numbers of recorded inscriptions to develop an index of functional diversity. Our results are consistent with theoretical expectations, adding further support to the view that urban systems in both contemporary and pre-modern contexts reflect a common set of generative processes.

## 1. Introduction

Over the last decade, researchers investigating contemporary urban systems have developed an integrated approach to the study of settlements, ‘settlement scaling theory’, which is grounded in a view of settlements as social networks embedded in built environments [1–4]. The fundamental process at the core of this framework is the concentration of interactions between individuals in space and time, albeit subject to a variety of constraints imposed by environmental conditions, technology and institutions [5,6]. The empirical hallmarks of this conception of settlements are systematic socio-economic effects induced by population size (scale) and population density for settlements in a given system [7–10]. This framework accounts for a series of patterns that have been identified in a number of settings spanning different geographical regions and chronological periods, including: (i) a consistent densification effect, such that larger settlements take up less area per person on average; (ii) intensified use of infrastructure, such that larger settlements use less material per person, again on average; (iii) increasing returns to scale in a variety of socio-economic outputs, including both measures of wealth and invention or innovation (measured through average GDP *per capita* and numbers of patents), but also crime, pollution and infectious disease; and (iv) increasing levels of functional diversity, such that larger settlements generally support a greater range of occupations [1,5,11].

Although the formal models that underlie settlement scaling theory can account for the attributes of contemporary urban systems, the mechanisms animating these models are very general and are not tailored to the specific conditions of the modern world or restricted to settlements of a certain size. This raises the question of the extent to which patterns observed in contemporary urban systems are also characteristic of pre-modern systems. To date, a number of studies using historical and archaeological data from a variety of pre-modern contexts have found evidence for several of the patterns outlined above, including the densification effect, intensified use of infrastructure, and increasing returns to scale [12–15]. This includes cities in the Roman Empire, where we have demonstrated that there is a relationship between the inhabited areas and densities of settlements that can be used to improve on estimates of their numbers of inhabitants [16]. Here, we extend this line of research by showing that these settlements also exhibit the same patterns of functional diversity with respect to population size observed in contemporary systems. Our results add further support to the view that throughout history human settlement systems have shared a common set of fundamental generative social processes which have led to consistent empirical patterns in their aggregate properties.

In this paper, we present a general model for the relationship between the *population size* and *functional diversity* of individual settlements within an urban system. Functional diversity is in turn interpreted as an indicator of the division of labour. We then explore the applicability of this model to pre-modern contexts, focusing on cities in the Roman Empire. To do this, we draw on current evidence regarding the numbers of inhabitants in these settlements and use existing information concerning the numbers of professional associations (akin to guilds) documented in inscriptions from each settlement to develop an index of functional diversity appropriate for this context. We then analyse the relationship between functional diversity and settlement population to assess the degree to which empirical patterns are consistent with theoretical expectations. Finally, we consider the implications of our results for the broader effort to develop a general approach to human societies as social, infrastructural, and wealth- and creativity-generating networks embedded in built environments.

## 2. The division of labour

The starting point of our analysis is Adam Smith's famous statement that ‘the division of labour is set by the extent of the market’ [17,18]. The standard interpretation of this observation is that larger markets support larger levels of production which, in turn, demand increasing separation of this production into discrete components and the increasing concentration of individuals on specific tasks [19]. A richer interpretation of this statement, which is not restricted to market economies, is that the extent of the division of labour is related to the number of people who interact with each other in pursuit of their livelihoods. Indeed, the relationship between population size and diversity of tasks, tools, and work done has been of interest to anthropologists and sociologists for decades [20–22].

At the level of individuals, specialization, broadly understood, implies performing fewer tasks while having to rely on others for the fulfilment of basic necessities and attainment of luxuries. Following Bettencourt and others [11], we propose that the range of tasks that an individual performs is inversely proportional to their social contacts, by which we mean all connections through which an individual can obtain goods or services. Consider that in a given context there is a range of ‘functions’ (tasks that need to be performed to fulfil certain ends) that each person must either perform themselves or have access to through others in order to survive. An isolated individual would have to perform all of these functions themselves, in which case the range of functions that they perform, *d*, would be equal to the total number of functions that need to be performed as a whole, *F*. In the case of settlements, however, we would expect individuals to have access to these functions through others via their social contacts. As an individual's social contacts *k* increase, the range of functions that a person must perform, or their *functional diversity*, can, without loss of generality, decrease proportionately [11]. Because most regular social contacts through which goods and services are exchanged are local, an individual's social contacts can be expected, on average, to be related to the size of the settlement in which they live. As a result, the relationship between the number of social connections, functional diversity, and the size of each settlement can be expressed as:
1.1
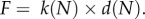
The relationship in equation (1.1) can be simplified by noting how an individual's social contacts *k*(*N*) should change with the size of the settlement [6]. In a fully-connected network, the total number of (bi-directional) links through which goods and information flow between individuals is *N* × (*N* − 1), which is essentially *N*^2^ for large *N*, implying *K*(*N*) = *N*^2^. However, for a social network embedded in space individuals are limited by the (energetic) cost of movement, as well as other implicit transaction costs entailed when interacting with others, such that only a fraction of the total potential connections are possible in each instance. If one further assumes that the population of a settlement is distributed homogeneously within the settlement area, *A*, then the total number of interactions that are possible per unit time is given by the portion of this area that a person explores per unit time. We represent this explored area as *a*_0_*l*, where *l* is the length of a person's path and *a*_0_ is a width representing the distance at which interactions occur. Putting these assumptions together we can write the following expression for settlement connectivity:
1.2
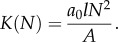
Note that the area taken up by a settlement can also be written as a function of the settlement population. Both theoretical and empirical considerations allow us to express the area taken up by a population of size *N* as:
1.3
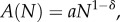
where *a* is a baseline area per person and *δ* reflects the rate at which the population density of the settlement increases with population. The value of *δ* ranges from 1/3 to 1/6, depending on the degree to which settlements are defined in terms of circumscribing areas versus built-up areas [1,14]. From here, one can substitute equation (1.3) into equation (1.2) and simplify, leading to:
1.4
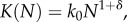
where *k*_0_ = *a*_0_*l*/*a* is a baseline level of connectivity. Now, given that the average connectivity per person is *k*(*N*) = *K*(*N*)/*N*, one can substitute this relation into equation (1.1) and simplify:

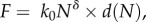

1.5
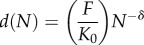

1.6
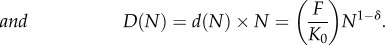


Equation (1.5) proposes that, on average, the functional diversity of an individual decreases with population at the same rate as the population density increases. Because functional diversity is the reciprocal of the division of labour, this relationship implies that the division of labour increases at this same rate. In addition, equation (1.6) suggests that the total functional diversity of a settlement increases more slowly than population and at the same rate as inhabited area increases. These relationships indicate that the population of the settlement will expand faster than its total functional diversity; but the overall division of labour will still expand, such that the number of distinct tasks performed by the population will increase [11]. It is important to stress, however, that these relationships will be a reflection of average conditions, since there will always be fluctuations in the exact number of contacts and range of functions from individual to individual and from settlement to settlement due to a variety of geographically- and historically-contingent factors.

Note that there is also a relationship between the division of labour and productivity. Typically, the gains following from an enhanced division of labour are attributed to the energy saved by increasing the intellectual and manual deftness of each worker through ‘learning by doing’, and by reducing the number of times individuals have to switch between tasks [23]. As a result, if the division of labour derives from levels of social connectivity, and this connectivity increases with population density, one would expect economic outputs to follow suit; such that if total social connections are given by *K*(*N*) = *k*_0_*N*^1+^*^δ^*, then total economic rates are given by *Y*(*N*) = *y*_0_*N*^1+^*^δ^*.

Studies of the division of labour tend to recognize two different forms of division: horizontal and vertical. The first normally refers to the diversity of activities related to production and exchange in an economy; whereas the second typically refers to the organization into different tasks within specific activities (or crafts and trades) [24]. Although this distinction is useful for some purposes, here we emphasize that horizontal and vertical divisions are actually related. As settlements grow in population, individuals tend to concentrate on a narrower range of tasks, even as the overall set of possible socio-economic tasks expands. Individual-level specialization presupposes and in turn induces specialization at the level of production, transportation, and distribution of goods and services (a distinction not restricted to modern economies). Due to these relationships, and the fact that functional diversity and division of labour are opposite sides of the same coin, it is feasible to measure the total functional diversity of a settlement in terms of the total number of tasks within the community. We apply this logic here in a study of the division of labour in settlements in a pre-modern context, in this case cities in the Roman Empire.

## 3. Definitions, focus and limits

The results and analysis presented here presuppose the identification of cities in the Roman Empire, and this assumes an answer to the seemingly straightforward query of what constitutes a ‘city’? In reality, answering this question is difficult even for contemporary societies. One influential definition was offered by the sociologist Louis Wirth [25] who noted that a city is a permanent settlement of heterogeneous individuals. Architectural historian Spiro Kostof observed that ‘cities are places where a certain energized crowding of people takes place’ [26, p. 37]). And the urban economist Edward Glaeser describes cities as ‘the absence of physical space between people and companies. They are proximity, density, closeness' [27, p. 6]. These characterizations encompass the perspective, prevalent among many who study contemporary urbanism, that the essence of urban life is frequent and intense social interactions among a diversity of individuals and institutions. Settlement scaling theory is similarly premised on seeing cities and settlements across the whole of the urbanization experience as social networks embedded in built environments.

Operationalizing a view of cities as settings for social interactions, which is to say assembling a set of spatial units of analysis which capture the relevant social aspects of settlements, requires choices about the use of existing data, the assignation of data to locations and periods, and the delineation of the spatial boundaries of inhabited areas, all of which are far from trivial even for data-rich modern urban systems [7]. When identifying cities, archaeologists and historians must perforce rely on textual sources and archaeological material derived from surveys and excavation to infer the social attributes of ‘energized crowding’.

To identify and characterize Roman cities, we have followed the definition used by Hanson in his recent account of the urbanism of the Roman world in the Imperial period [28]. As he notes, although it is notoriously difficult to define urbanism, one can come up with a working definition by concentrating on sites that are more likely to have engaged in secondary and tertiary activities than primary activities, and this can be gauged by whether they had a certain population (such as 1000, 5000 or more individuals) or offered certain non-subsistence functions (such as historical, social, cultural, religious, political, administrative, juridical and economic roles). Although we do not have direct evidence for these features, we can approximate them by looking at the size of inhabited areas, monumentality and civic status in ancient sources. This provides us with a number of criteria, which include not only whether sites conform to thresholds of 10 or 50 hectares (a reflection of their numbers of inhabitants), but also whether they had monuments, such as public spaces, associated public buildings, urban grids, leisure and entertainment structures, and religious, sanitary, and defensive structures, and whether they had civic statuses, such as roles as provincial capitals, *conventus* capitals, *metropolis* capitals, *nome* capitals, *coloniae*, *municipia*, *civitates*, and *poleis*, or various other rights and privileges. These features do not necessarily coincide, as there are a small number of sites that do not meet the criteria for size, but nonetheless have significant monumentality or civic status. Due to these complexities, we have restricted our investigation to the catalogue of cities considered by Hanson based on the criteria above. This catalogue encompasses the region covered by the Roman Empire at its maximum extent in A.D. 117 and the period between the first century B.C. and the third century A.D.

## 4. Material and methods

**Figure 1.**
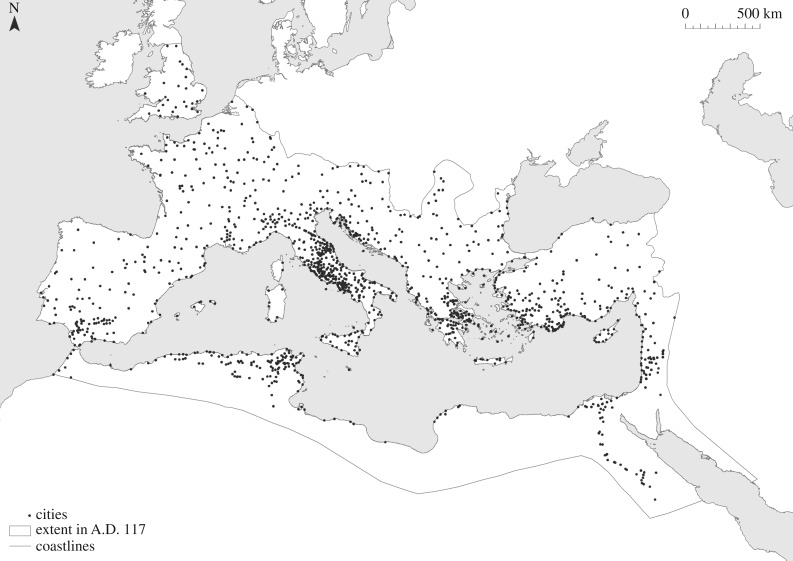
The cities of the Roman world in the Imperial period, adapted from [28].

We use three different datasets to examine the relationship between urban populations and their levels of functional diversity. The first is Waltzing's lists of associations, usually known as *collegia*, which identify the number of distinct craft and trade organizations that are known to have been active in a given settlement [29]. The second is the total number of inscriptions recorded for each settlement in the Epigraphik-Datenbank Clauss/Slaby (http://db.edcs.eu/epigr/epi.php?s_sprache=en, accessed 19 January 2017), which we use to characterize the amount of material from which Waltzing's lists derive. The third is Hanson's catalogue of cities and towns in the Roman world in the Imperial period (figure 1; described in more detail below), which not only includes information about their locations and date ranges, but also evidence for the size of their inhabited areas [28]. These can be converted into estimates of the numbers of inhabitants in these settlements based on densification effects [16]. We combine these three data sources to create an index suitable for testing the expectations of settlement scaling theory regarding the relationship between population and functional diversity. Below we discuss the details surrounding each data source.

### 4.1. Associations

Although there has been a lot of work done on occupations in the ancient world, such studies have tended to focus on examining the range of occupations across settlements rather than instances of specific occupations in specific settlements. As a result, although it is feasible to count the total number of occupations, it is not feasible to determine which ones occurred where. This issue stems from a tension between the sheer mass of evidence that might provide references to occupations, such as texts, inscriptions, *papyri*, and even *graffiti* and *dipinti*, versus the disconnected nature of historical and archaeological research that has been done on individual sites, regions, or classes of material. As a result, scholars are able to identify around 700 occupations for the Roman Empire as a whole, but are only able to count the numbers of occupations in specific settlements in a handful of cases, such as Rome and a few other sites [24,30–32].

Although it is not currently possible to quantify individual occupations across settlements, it is possible to quantify functional diversity at a more general level by tracking the number of *associations* mentioned in various sources, most notably inscriptions. These associations were voluntary organizations of craftsmen or traders that were referred to using various terms, the most familiar of which is *collegia* [33]. They were modelled after the local governments of cities and towns; had their own magistrates, councils, and assemblies; and even had their own premises and treasuries. Associations were open to nearly all classes of men (including slaves and ex-slaves), but did not allow women or children. Having said this, some associations were more influential than others, leading to intense competition between associations for status, as well as to the setting up of alliances between associations or to the drawing of distinctions among their own members. In addition, associations might have had an important function in helping to orientate newcomers to settlements, including helping them find colleagues, source materials, share labour, and identify customers, so some scholars have seen the growth of associations as a symptom of the boom of urban life, the expansion of settlements, and their reliance on migration to maintain or increase their numbers of inhabitants [34]. Overall, associations were a conspicuous feature of settlements that played an important role in the social life of the community [35].

There has been significant debate among classical archaeologists and ancient historians concerning the extent to which associations were intended to foster or defend their members' economic interests. The traditional view has been that these bodies were mainly set up for social reasons and had limited economic consequences [36]. However, in recent years there has been a shift in opinion and an increasing appreciation of the roles of institutions in shaping economies under the influence of New Institutional Economics [37–39]. This work has emphasized the extent to which associations created networks of trust, which were only feasible because of their closed nature, internal traditions, and enforcement mechanisms built on the status and reputations of their members [33]. One would therefore expect these networks to have had economic implications, since they helped to strengthen alliances between members, disseminate information, and lead to the sharing of knowledge and skills.

As a result, most recent scholars have emphasized the multi-dimensional roles of these associations, including: attaining and maintaining social standing; enhancing status and demonstrating wealth; taking part in convivial activities such as drinking and feasting; offering surrogate familial environments to orphans, foreigners, and resident aliens; observing religious rituals, ceremonies, and festivals; ensuring that members had a suitable burial and looking after their memories (such as maintaining their tombs or performing certain rituals after their death); taking part in group attendance at events (although any suspicion of incitement was quickly supressed); offering legal rights and privileges; and perhaps extending financial assistance to their members. There is also evidence that associations were involved in the following areas: the arrangement of collective work; control of wages; organization of strikes; creation of monopolies; management of their own funds; extension of loans; inhibiting competition; regulating prices; creating and enforcing weights and measures; and taking care of the election and training of apprentices [40]. Based on this work, we expect most crafts and trades to have formed an association, meaning that it is reasonable to treat association diversity as a proxy for the overall diversity of socio-economic activities that occurred within settlements.

**Figure 2.**
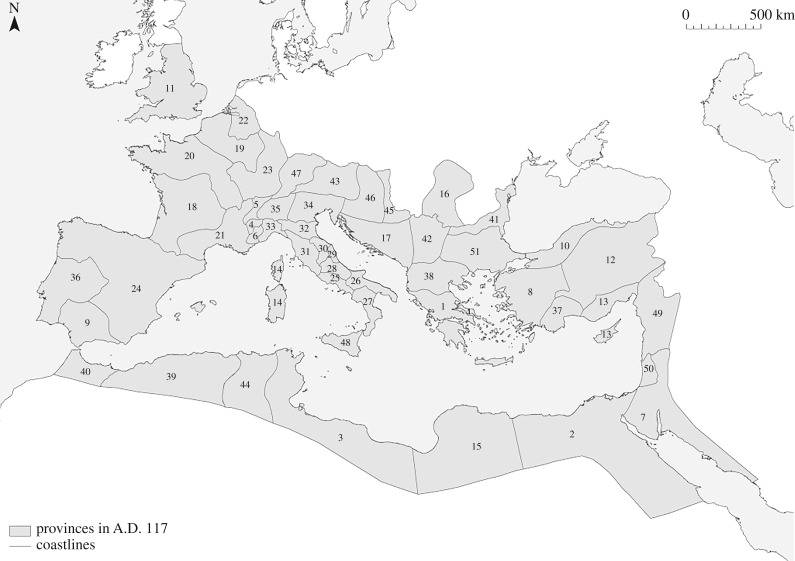
The provinces of the Roman Empire at the death of Trajan in A.D. 117, adapted from [28]. 1: Achaea; 2: Aegyptus; 3: Africa Proconsularis; 4: Alpes Cottiae; 5: Alpes Graiae et Poeninae; 6: Alpes Maritimae; 7: Arabia; 8: Asia; 9: Baetica; 10: Bithynia et Pontus; 11: Britannia; 12: Cappadocia et Galatia; 13: Cilicia et Cyprus; 14: Corsica et Sardinia; 15: Creta et Cyrenaica; 16: Dacia; 17: Dalmatia; 18: Gallia Aquitania; 19: Gallia Belgica; 20: Gallia Lugdunensis; 21: Gallia Narbonensis; 22: Germania Inferior; 23: Germania Superior; 24: Hispania Tarraconensis; 25: Italia (I Latium and Campania); 26: Italia (II Apulia et Calabria); 27: Italia (III Lucania et Brutii); 28: Italia (IV Samnium); 29: Italia (V Picenum); 30: Italia (VI Umbria and Ager Gallicus); 31: Italia (VII Etruria); 32: Italia (VIII Aemilia); 33: Italia (IX Liguria); 34: Italia (X Venetia et Histria); 35: Italia (XI Transpadana); 36: Lusitania; 37: Lycia et Pamphylia; 38: Macedonia; 39: Mauretania Caesariensis; 40: Mauretania Tingitana; 41: Moesia Inferior; 42: Moesia Superior; 43: Noricum; 44: Numidia; 45: Pannonia Inferior; 46: Pannonia Superior; 47: Raetia; 48: Silicia; 49: Syria; 50: Syria Palestina; 51: Thracia.

There are two concerns, however, that need to be addressed before using associations in this manner. The first is whether the epigraphic record evidence concerning associations is more or less abundant than evidence concerning specific occupations. We expect references to the former to be preserved more frequently than the latter due to the relative size, status, and wealth of associations; and the fact that associations regularly set up identifiable memorials for their deceased members. Moreover, even if associations were only related to certain sectors of the local economy, association diversity should still be a reasonable proxy for relative functional diversity across settlements. The second issue is whether evidence for associations is consistently preserved across the length and breadth of the Roman Empire. The available information concerning associations is clearly structured by affordances, such as divergences in the epigraphic habits of different times and places (as a result of differences in wealth, education, fashion, local language, acculturation, etc.), levels of preservation, rates of recovery, and levels of investigation by historians and archaeologists. Here, we control for these factors by relating the number of associations identified for each settlement to the number of inscriptions that have been studied, and by standardizing our index of association diversity by imperial provinces (figure 2).

To estimate the numbers of associations in individual settlements we have relied mainly on Waltzing's *Étude historique sur les corporations professionnelles chez les Romains* [29], which was considered ground-breaking when it was originally issued between 1895 and 1900 and which is still regarded as the standard work on associations even now. The most relevant information is contained in one detailed list of associations in Rome, Ostia, and Portus and another for the other cities and towns, totalling 802 references to associations across 250 settlements ([29], volume IV: 4–49 and 49–128, along with volume II: 145–157). However, since these lists were mainly based on epigraphic material, it is somewhat skewed towards the west rather than the east. In addition, there is also some information about the numbers of more informal bodies (which are usually called *societas*), as well as associations that had an overtly religious or military character. We have not included these because they do not relate to crafts and trades.

**Figure 3.**
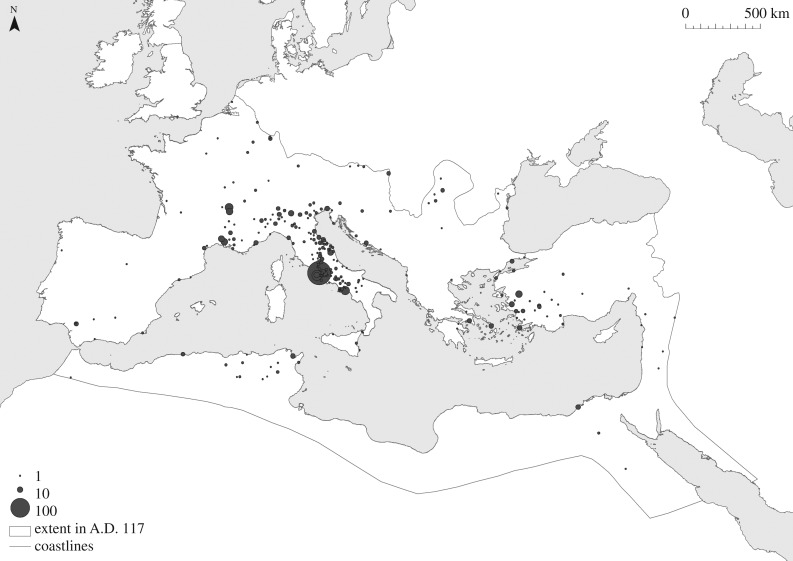
The numbers of associations in cities in the Roman world during the Imperial period.

Although there is an ongoing attempt to update Waltzing's database of associations by other researchers, it will be some time before these new resources are available. In the meantime, we have attempted to deal with the most serious issues surrounding Waltzing's data [41–47], but have not reviewed them in detail. We have divided references to associations whose titles encompass more than one craft or trade into separate references for each one (of which the most common are *fabri*, *centonarii* and *dendrophori*). The resulting dataset is displayed in figure 3.

### 4.2. Inscriptions

One would expect the number of associations that have been identified in each settlement to be a function, not only of the underlying functional diversity of that settlement, but also the amount of material that has been preserved, recovered and examined from that settlement. To control for these factors, we used the most comprehensive online resource currently available, the Epigraphik-Datenbank Clauss/Slaby (which at the time of writing contains over 500 000 entries), to tabulate the total number of inscriptions recorded for each site. It should be noted that this resource only includes texts in Latin, rather than in Greek (or any other languages), meaning that it is also skewed towards the west rather than the east. Since it is difficult to link each inscription to a specific settlement using a name or a region, we linked inscriptions to settlements by associating the find spot of each inscription to the nearest settlement using their coordinates and a 5 km buffer in a GIS. The size of the buffer reflects the relative accuracy and precision of the coordinates for both these settlements and the find spots of inscriptions. The Epigraphik-Datenbank Clauss/Slaby is an active database that includes more inscriptions than Waltzing had access to, and as a result the relationship between the Waltzing associations count and the Clauss/Slaby inscription count is approximate. Having said this, the ratio of associations to inscriptions should provide a better sense of the diversity of associations in a given settlement than the raw count of associations with no attempt to control for sample size (see below). We therefore divide the number of associations by the number of inscriptions for each settlement, generating a ratio, *R*, which effectively provides a measure of the diversity of associations per inscription. There are clearly errors between the sample ratios of associations to inscriptions and their actual, but unknown, population ratios. We would expect these errors to be independent of the populations of settlements, however, such that they would influence the dispersion of the data around the central tendency of the relationship as opposed to changing the relationship between population and functional diversity itself.

### 4.3. Sizes and populations

To estimate the sizes and populations of ancient settlements we have drawn on existing estimates of their inhabited areas. These estimates are based on a number of features, including the area enclosed by walls, the extents of urban grids, the locations of monumental structures, the sizes of residential zones, the situation of cemeteries, and even the character of natural features, such as changes in relief and the courses of rivers and coastlines. We then incorporate our recent work on the average relationship between inhabited area and population density in Greek and Roman settlements to convert these areas into population estimates [16]. To establish this relationship, we counted the number of residential units in excavated areas in a selection of settlements and combined this with the average size of a household (which we assumed averaged about 5) to estimate the population density of each excavated area. Using this approach, we were able to estimate the population density of 52 sites, which are scattered throughout the settlement hierarchy and across the Greek and Roman world from the fourth century B.C. to the fourth century A.D.

**Figure 4.**
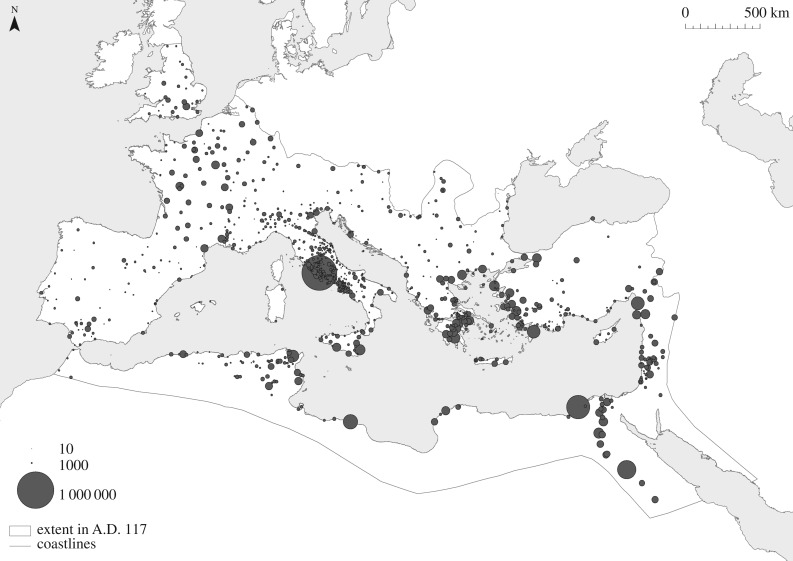
The estimated numbers of inhabitants in cities in the Roman world during the Imperial period, after [16].

This material suggests there is a strong relationship between the population density and inhabited areas of these settlements. The parameters of this relationship are consistent with the expectation of settlement scaling theory that the population *N* of a settlement should expand with settled area *A* according to *N* = *dA*^1/^*^α^*, where *d* is the (baseline) population density of the smallest settlements in the sample and 2/3 ≤ *α* ≤ 5/6 [14]. In addition, population estimates deriving from this relationship accord well with the small number of sites where we can gauge population using other means [16]. The result of this work is a regression equation that allows one to estimate the number of inhabitants in an ancient settlement from its built-up area. This can be expressed as:
4.1
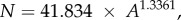


where *N* is the number of inhabitants, 41.834 is the baseline population density in people per hectares, and *A* is the inhabited area in hectares (*p* < 0.0001, *r*^2^ = 0.847). It is important to note that these estimates differ slightly from those in Hanson [28], as the latter are based on density classes correlated with size classes, rather than discrete figures for each site. Also note that this relationship implies that settlements grew denser, on average, as their built areas increased. We use these population estimates (displayed in figure 4) as the independent variable in the analyses that follow.

## 5. Index of functional diversity and estimation framework

The data discussed above provide evidence for a total of 802 associations (range = 1 to 155, average = 3) in 250 sites distributed throughout the Roman Empire. However, since inhabited areas (and therefore populations) and/or inscription totals are not available for all settlements, the dataset with no missing values includes information for 210 settlements. The resulting dataset is available as electronic supplementary material, as well as at: http://core.tdar.org/project/392021/social-reactors-project-datasets. For each settlement in our analysis we have a total number of distinct associations, a total number of inscriptions that have been documented, and an estimated population. We assume that all three values reflect average conditions during the occupation of each settlement, and that inscriptions accumulated for comparable lengths of time across settlements.

**Figure 5.**
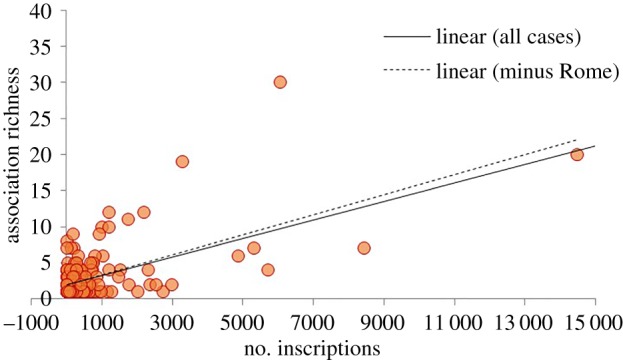
The relationship between inscription count and association diversity in the analysis dataset. The data for Rome (119 532 inscriptions, 155 associations) are beyond the range of the display; the fit lines show the effect of removing Rome from the analysis (for all data, *y* = 0.0013*x* + 1.9609, *r*^2^ = 0.9426; excluding Rome, *y* = 0.0014*x* + 1.8969, *r*^2^ = 0.3607; *p* < 0.0001 in both cases).

We develop an index of functional diversity for these settlements in two stages. First, we divide the observed association diversity by the total number of inscriptions to yield the ratio *R* of association diversity per inscription. This measure is analogous to the concept of *species density* in ecology (the number of distinct species observed per area). This measure has been shown to be problematic in an ecological context due to the asymptotic nature of species–area curves, which imply that the probability of obtaining a previously unobserved species declines as sampling intensity increases [48]. We do not have access to raw counts of references to each association type for specific settlements and as a result we are unable to test this possibility directly using rarefaction or analogous methods [49]. However, the relationship between inscription count and association diversity in the dataset does not show a pattern of asymptotic increase. Instead, the fit line that best captures the relationship is a linear function, even when data for Rome are excluded (table 1; figure 5). This suggests the probability of encountering an additional association type does not decline with sample size in these data. We suspect the reason for this is that associations are only mentioned in a small fraction of inscriptions. As a result, the small probability of drawing an inscription that mentions an association plays a much larger role than the probability that one of these will be a duplicate reference in producing the observed pattern. Given this, it is reasonable to divide the number of distinct associations mentioned by the number of inscriptions examined to generate a measure of association diversity density that controls for sample size.

**Table 1.** The relationship between inscription count and association diversity in the analysis dataset.^a^

fit type	*R*^2^
linear	0.943 (0.361)
exponential	0.198 (0.203)
logarithmic	0.118 (0.134)
power	0.169 (0.125)

^a^All regressions are significant at the *p* < 0.0001 level; values in parentheses reflect regressions that exclude Rome; the sample size is 210 settlements (209 when Rome is excluded).

Second, we assume that, because most inscriptions were memorials for or dedications to the achievements of specific persons, the number of inscriptions available for a given settlement is a measure of the number of people commemorated over time in that settlement. This in turn implies that the ratio *R* is proportional to the functional diversity *per capita*, or *d*(*N*), for that settlement. Thus, one can multiply this ratio by the number of inhabitants in each settlement to yield a measure proportional to *D*(*N*), the total number of associations that existed in a given settlement during its period of peak occupation. Ultimately, what we are interested in is the statistical relationship between *D*(*N*) and the population *N* across settlements, as represented by equation (1.6) above. However, due to the fact that we estimate total functional diversity as *D*(*N*) = *d*(*N*) × *N*, the population variable is involved in the creation of the dependent variable as well as playing the role of an independent variable. The multicollinearity effects introduced by this procedure makes it imperative to confirm that functional diversity *per capita*, *d*(*N*), also relates to population as predicted by equation (1.5). The discussion below describes the regression estimation framework used to arrive at these desired statistical destinations.

The data used in this investigation are for settlements located in different areas of the Roman Empire. Although it is meaningful to describe this entity as a single system, in which settlements were linked via political, administrative, juridical, fiscal and military interactions, it is also clear that there were differences between each region, which modulated the relationship between settlement scale and functional diversity. It is well-known that the so-called ‘epigraphic habit’ was stronger in Latin-speaking provinces than in Greek-speaking provinces [50], and that associations were more important in Latin-speaking than Greek-speaking regions. Since the data sources we use focus on Latin rather than Greek inscriptions, one would expect the relative use of Latin and Greek to have affected the underlying rate of inscription production and the rate at which associations are mentioned in these inscriptions. Also, it is likely that the inscription production rate was related to other underlying social and economic conditions, such as differences in the distribution of wealth, the spread of literacy, or deference to customs in different regions, which were in part a result of the length of time each region was incorporated into the Roman Empire.

One would expect these factors to introduce heterogeneity to the statistical relationship between functional diversity and population size at the settlement level. For this reason, we use a fixed-effects estimation framework using the imperial province that each settlement was located within as a control variable (since these varied, we used the imperial boundaries on the death of Trajan in A.D. 117). This controls for geographical and chronological variation, as well as the degree to which Latin or Greek was spoken in each region, because provincial boundaries reflect the history of imperial expansion. This procedure for obtaining location-specific counts is similar to the ‘Empirical Bayes Adjustment’ method often used in epidemiological studies to generate place-specific counts of infected individuals on the basis of small samples of infection rates [51,52]. All estimations were obtained using the AREG routine, which assigns a dummy variable to each province, controlling for heteroscedasticity, in Stata version 12SE. Other fixed-effects estimation methods yielded similar results.

We estimated four equations, regressing the natural logarithm of the dependent variable against the natural logarithm of settlement population, *ln* (pop), each of which generates a result of interest in its own right, with the first three equations as steps along the way to the fourth and most important result:
5.1
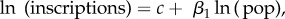

5.2
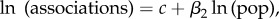

5.3
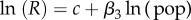

5.4
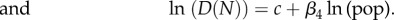
Concerns that equation (5.4) includes the variable for population on both sides of the equals sign (given how the functional diversity measure is calculated) are assuaged by noting that the ratio of associations to inscriptions is specific to each settlement. Multiplying the ratio by settlement population produces an estimate of an aggregate count; equation (1.6) postulates a systematic relationship between this count and population across the Roman Empire. We hasten to clarify that the goal of the regression exercise is not to postulate econometric models which can account for a large proportion of the observed variability in the dependent variable. Rather, the simple regression models serve as the means to assess the empirical validity of a specific expectation, via a predicted coefficient, regarding how the dependent variable should scale with population.

## 6. Results

Our estimation results are presented in table 2. In all four analyses the scaling coefficient is statistically significant at the 95% confidence level. The relationship between settlement population and total inscriptions suggests the rate of increase in the inscription rate with population was comparable to the rate of increase of inhabited area with population (0.643 for inscriptions versus 0.634 for area). This implies that inscriptions were generated proportionately to the settlement area, not necessarily population, and that one might therefore interpret inscriptions as a sort of information infrastructure in ancient cities. In this scenario, the inscription viewing rate would be proportional to the population density, such that each inscription was viewed more frequently as settlement population and density increased. As a result, fewer inscriptions *per capita* were needed for the information that they contained to percolate through the settlement.

**Table 2.** Analysis results.

dependent variable	inscriptions	associations	associations/inscriptions	*D*(*N*)
intercept	−0.341	−2.147	−1.807	−1.821
*β*	0.643 (0.092)	0.328 (0.048)	−0.314 (0.087)	0.657 (0.077)
95% CI	[0.461, 0.825]	[0.233, 0.424]	[−0.486, −0.141]	[0.614, 0.797]
*R*^2^	0.58	0.35	0.59	0.66
*N*	210	210	210	210

The relationship between functional diversity *per capita* and population is also important in that it shows that the ratio of associations to inscriptions, which we take to be a measure of *d*(*N*), declines with settlement population in accordance with the expectations of settlement scaling theory. Specifically, given that the point estimate for the value of *δ* for the Roman Empire is 0.314, based on patterns in the density of residential units, one would expect *d*(*N*) to decrease at this same rate as the settlement population grew. This is in fact what we observe, to within a single standard error of the estimate. Note also that the *R*^2^ value of this relationship is reasonably high despite the many sources of noise affecting the data.

Finally, our index of total functional diversity in ancient settlements, which we calculate as the total number of associations divided by the total number of inscriptions, multiplied by the total population, also scales with population in ways that are predicted by theory. Specifically, the coefficient of this relationship indicates 1 − *δ* = 0.657, and thus that *δ* = 0.343*.* This point estimate is once again within a single standard error of the value for *δ* estimated from the density of residential units. Note that population size alone can explain upwards of 60% in the variability across settlements of functional diversity. (The estimated value for the scaling coefficients are not much changed when Rome, the largest city in the system by several orders of magnitude, is excluded from the observations.)

**Figure 6.**
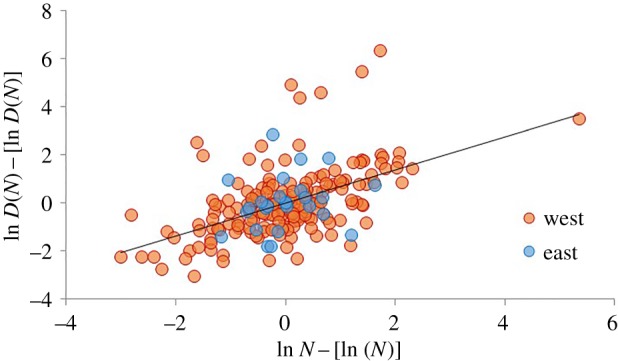
The relationship between population and functional diversity in ancient Roman cities. In this chart the data are centred by province by subtracting the mean from each value, following log transformation, see [7]. This procedure controls for variation in epigraphic habits across the Empire to some extent. Cities from the western provinces are shown in red and cities from the eastern provinces in blue. The slope of the best-fit line (all cases) is 0.686 (s.e. = 0.078, *p* < 0.0001, *r*^2^ = 0.2670).

Estimating scaling parameters for an urban system which spans different regions and periods can affect the exactness of the estimates, a concern addressed by controlling for imperial provinces when regressing the different dependent variables on population size. Another method for pooling data drawn from smaller settlement systems (based on imperial provinces), which can be expected *a priori* to have different baseline metrics, is to centre the data after log transforming it (see [7]), such that the data for each province have a mean of zero on both variables. Figure 6 illustrates the scaling relationships using this alternative procedure which leads to an estimate of 1 − *δ* = 0.686, (s.e. = 0.078) which is very similar in magnitude to the result obtained using fixed-effects modelling (although, not surprisingly, the *r*^2^ value is lower in this case).

In sum, despite the many sources of noise in our data, and the many assumptions we must make in turning these data into proxies for functional diversity and population, the relationships between them are all consistent with the theoretical framework presented in this paper, and thus provide empirical evidence that the relationships between settlement population and functional diversity observed in modern urban systems [11], and predicted by settlement scaling theory [5], are also apparent a pre-modern context—in this case, cities in the Roman Empire. Our results also add support to the assumptions we make in constructing our index of functional diversity, such as the notion that associations provide an index of functional diversity, and that inscriptions were made to honour the achievements of specific individuals and therefore are connected to demographic conditions.

## 7. Discussion and Conclusion

Although this analysis emphasizes the effects of city size for the division of labour, it should be emphasized that Roman cities did not exist in isolation but were linked to wider systems, hierarchies and networks. Ancient sources, for example, mention cities and towns that were ‘famous’ or ‘notable’ for certain commodities, which suggests the development of groups of complementary settlements [28]. We have not addressed these aspects of the urbanization and economic development, which reflect the development of central functions, comparative advantage, trade routes, and a variety of other factors. Our focus here is on the extent to which economic functions are divided up within a local community, and the role of local social connectivity in this process. Our framework suggests that the range of functions a given individual performs is inversely proportional to their social connectivity; that social connectivity increases with settlement density; and that density increases with settlement population. As a result, the range of functions performed by each individual declines with city size, even as the range of functions performed by the group overall expands. The results presented here add support to this framework.

Among ancient historians and archaeologists there is growing awareness that ancient economies were not stagnant, but experienced a great deal of change between 1000 B.C. and 1000 A.D., including extended periods of growth and decline. This has led to an increasing awareness of economic efflorescences in specific times and places, including Classical Greece and the High Roman Empire [53–56]. Still, questions remain about the nature and magnitude of this growth, how broadly its benefits were felt, what caused it, and how it compared to that of later eras. Our results contribute to this discussion by suggesting that the economic florescence of the Roman Empire derived at least partly from increased efficiencies in production deriving from an expanding division of labour facilitated by urbanization. To clarify this point it is useful to distinguish two kinds of economic growth: extensive (or aggregate) growth and intensive (or *per capita*) growth. The first is normally understood as being caused by increases in the factors (inputs) of production or by a simple increase in population, leading to an increase in the total amount of output generated by an economy. The second is usually regarded as being caused by an increase in the efficiency of production, so that each worker creates more goods or provides more services, leading to an increase in the total amount of wealth generated *per capita* [57]. This second type of growth can take one of two forms, often referred to as ‘Smithian’ versus ‘Promethean’ growth. The first results from specialization made possible by increases in the size of the market or the amount of trade, while the second type of growth is driven by the use of more energy-intensive fuel sources or by technological change [58]. In this context, our results suggest Roman cities were an important driver of ‘Smithian’ growth due to their ability to concentrate individuals in space and time and therefore enhance the opportunities for them to interact, share resources, and exchange skills, knowledge, and ideas.

It is also important to note the remarkable amount of urbanism that characterized the Roman Empire relative to preceding and subsequent periods [59]. This is witnessed by not only the maximum size of settlements, since Rome, with approaching a million inhabitants in the second century A.D., was not surpassed until London had the same number of residents in 1800; but also by the size of the urban population, which was at least 14 million (using an urban threshold of 5000 individuals) at this time, putting it on a par with Europe in the eighteenth century [60]. This pattern, in combination with our findings, reinforces the notion that economic development during the imperial period was systematically related to the growth and decline of urbanism in the same era.

In this paper, we have provided empirical support for the view that cities served as places of ‘energized crowding’ in ancient societies by demonstrating that levels of functional diversity in cities in the Roman Empire changed with settlement population, on average, in ways that are consistent with a theoretical expectation that unifies population, population density, social connections, division of labour, and economic outputs. Our results suggest that economies in a wide range of contexts, including in the past and present, evolved in accordance with a single set of social processes related to both the structure of human networks and the ways in which their properties change as the number of people who are connected by them grow. These results have important consequences for the scope of application of settlement scaling theory, since they not only add credence to the theory itself, but also add credibility to the idea that it applies broadly to both ancient and contemporary contexts [12–15].

Our results, if true, have significant implications for our understanding of the overall trajectory of urbanization and economic development over the very long run, since they suggest urbanism made an important contribution to economic development in both ancient and modern times. Although we do not have adequate data for a chronological analysis, our theoretical framework, and our results, imply that functional diversity did in fact change in accordance with the distribution of settlement sizes over time. There may also have been changes in baseline levels of functional diversity at the same time, but addressing this question will require more abundant or nuanced data than we have been able to marshal here. Also, despite the fact that we have not measured economic outputs directly, the fact that functional diversity scales with settlement population in a way that implies increases in social connectivity, and thus aggregate outputs, suggests that *per capita* economic outputs did change through time in accordance with changes in settlement size distributions. Indeed, our results suggest that, if one could track proxy measures of socio-economic outputs as well as inhabited areas through time, one should be able to reconstruct not only demographic trends, but also changes in aggregate outputs. We hope progress will be made in these areas in future work.

## Supplementary Material

Supplementary information

## Data accessibility

The data analysed in this article are available as electronic supplementary material, as well as at: www.tdar.org.
